# Upconversion photoluminescence excitation reveals exciton–trion and exciton–biexciton coupling in hBN/WS$_{2}$/hBN van der Waals heterostructures

**DOI:** 10.1038/s41598-022-18104-z

**Published:** 2022-08-11

**Authors:** Ewa Żuberek, Martyna Majak, Jakub Lubczyński, Joerg Debus, Kenji Watanabe, Takashi Taniguchi, Ching-Hwa Ho, Leszek Bryja, Joanna Jadczak

**Affiliations:** 1 Department of Experimental Physics, Wrocław University of Science and Technology, Wybrzeże Wyspiańskiego 27, 50-370 Wrocław, Poland; 2 Department of Physics, TU Dortmund University, 44227 Dortmund, Germany; 3 National Institute for Materials Science, Tsukuba, Ibaraki 305-0044 Japan; 4 Graduate Institute of Applied Science and Technology, National Taiwan University of Science and Technology, Taipei, 106 Taiwan

**Keywords:** Physics, Condensed-matter physics, Condensed-matter physics, Materials science, Two-dimensional materials, Nanoscience and technology, Nanoscale materials, Two-dimensional materials

## Abstract

Monolayers of transition-metal dichalcogenides with direct band gap located at the binary $K_{-}/K_{+}$ points of the Brillouin zone are promising materials for applications in opto- and spin-electronics due to strongly enhanced Coulomb interactions and specific spin-valley properties. They furthermore represent a unique platform to study electron–electron and electron–phonon interactions in diverse exciton complexes. Here, we demonstrate processes in which the neutral biexciton and two negative trions, namely the spin-triplet and spin-singlet trions, upconvert light into a bright intravalley exciton in an hBN-encapsulated WS$_{2}$ monolayer. We propose that the energy gains required in the polarized upconversion photoluminescence originate from different interactions including resonant optical phonons, a cooling of resident electrons and a non-local and an anisotropic electron–hole exchange, respectively. The temperature dependence (7–120 K) of the excitonic upconversion intensity obtained at excitation energies corresponding to the biexciton and trions provides insight into an increasing phonon population as well as a thermally enhanced electron scattering. Our study sheds new light on the understanding of excitonic spin and valley properties of van der Waals heterostructures and improves the understanding of photonic upconversion mechanisms in two-dimensional quantum materials.

## Introduction

Two-dimensional (2D) materials have attracted considerable scientific attention in the last decade. In particular, transition-metal dichalcogenide (TMDC) monolayers, such as MoS$_2$, MoSe$_2$, WS$_2$ and WSe$_2$, have emerged as a semiconducting alternative to the zero-band-gap graphene. The atomically thin TMDCs possess direct band gaps in the visible and near-infrared ranges at the $K_{-}$ and $K_{+}$ points of the Brillouin zone^1^. The two-dimensional confinement of charge carriers in TMDC monolayers leads to a reduced dielectric screening of the Coulomb interaction and a formation of excitons with binding energies of hundreds of meV^1,2^. Due to a strong spin-orbit coupling in the TMDC monolayers and a resulting valley-contrasting spin splitting of the energy gap at the $K_{-}/K_{+}$ points, the electrons and holes possess both the spin and valley degree of freedom^3^. Thus, the TMDCs exhibit unique magneto-optical and opto-electronic properties which are very promising for applications in spin- and valleytronics^2,4,5^.

In addition to neutral excitons, a manifold of exciton complexes may be formed with binding energies of tens of meV^6–8^; however, their identification in optical spectra was beyond the reach of simple structures exfoliated directly on standard SiO$_2$/Si substrates^9^. Recent progress in elaborating high-quality van der Waals (vdW) heterostructures based on hBN-encapsulated TMDC monolayers allows for observing the subtle optical properties of the exciton complexes thus offering the exploration of many-body effects including electron–electron and electron–phonon interactions^10–17^. For W-based vdW structures, electron–electron interactions manifest themselves in a trion photoluminescence (PL) line splitting into an inter-valley triplet and intra-valley singlet whose energies are separated by about 6 meV^6,7,18^. Alternatively, the electron–electron coupling may yield the observation of the biexciton fine structure^19^. Instead, electron–phonon interactions give rise to a coupling between the ground and excited states of the neutral exciton realized by an exchange of phonons in a Raman scattering process^20^.

Moreover, photonic upconversion (UPC) is a highly relevant phenomenon of light-matter interaction which not only is studied in fundamental sciences like physics and chemistry, but also finds application in fluorescence UPC in optical fibers^21,22^. The optical UPC is a fundamental light–matter interaction, an anti-Stokes-like process, in which an absorbed photon receives energy from the environment and is re-emitted at an energy higher than the excitation energy. Different mechanisms are known for describing UPC in different material systems; however, the mechanisms of exciton UPC in particularly 2D quantum materials are not well understood^23^. In that context, an open question is related to the exciton emission upconverted from three- or four particle states which are addressed in the transparency window of the TMDC monolayer. Additionally, hitherto, only in WS$_2$ monolayers without encapsulating hBN layers, excitonic UPC processes were studied^11,14^.

In the current manuscript, we demonstrate the UPC of the exciton emission, for the first time, in hBN-encapsulated WS$_2$ monolayers which are characterized by narrow spectral properties (about 2–4 meV) compared to that of uncapped monolayers or monolayers placed on SiO$_2$. This spectral line narrowing allows us to unambiguously reveal the nature of the upconverting states and mechanisms. For resonantly addressing the coupling between different exciton complexes in the hBN-encapsulated WS$_2$ monolayer with low electron doping, we tune the laser excitation energy below the neutral exciton. This UPC photoluminescence excitation (PLE) measured at cryogenic temperatures reveals emission of the neutral intravalley exciton X upconverted from the neutral biexciton (XX$^{0}$) as well as the spin-triplet (T$_{\mathrm {T}}$) and spin-singlet (T$_{\mathrm {S}}$) trions. While the exciton emission upconverted from the trions is mainly based on spin-conserving electron–phonon and electron–electron scattering, the biexciton-mediated UPC is attributed to a non-local electron–hole exchange and an anisotropic (flip-stop) interaction. Furthermore, we evaluate the UPC dependence on changes in the temperature and the polarization degree of the exciting laser light. The polarization-resolved experiments indicate that the UPC PL is more efficient, for resonant excitation of the $K_{+}$ valley. Our study enhances the understanding of fundamental upconversion processes in vdW heterostructures and contributes to their application as opto-electronic new-generation devices.

**Figure 1:**
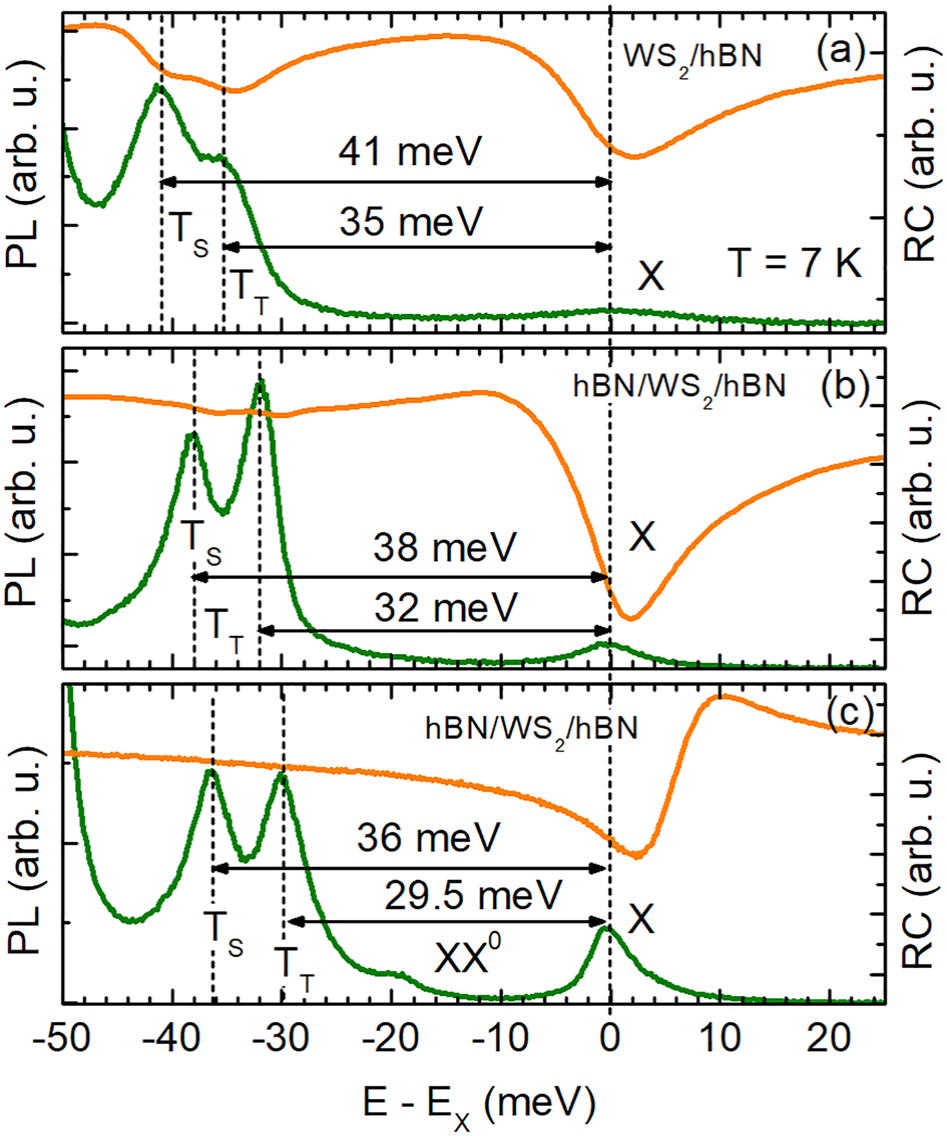
PL (green curves) and RC (orange curves) spectra of different vdW heterostructures assembled from a WS$_{2}$ monolayer and hBN layers, measured at 7 K. (**a**) s$_1$-WS$_{2}$/hBN with $d=240$ nm, (**b**) s$_2$-hBN/WS$_{2}$/hBN with $d=15$ nm and (**c**) s$_3$-hBN/WS$_{2}$/hBN with $d=240$ nm. The energy differences between the neutral exciton and the singlet and triplet trion PL lines are indicated in each panel.

## Results

### Photoluminescence and reflectivity of WS$_{2}$/hBN and hBN/WS$_{2}$/hBN heterostructures

Figure 1 compares the low-temperature photoluminescence (PL) and reflectivity (RC) within an energy range from the neutral exciton X to the trions for different WS$_2$-based heterostructures. In panel (a) the PL and RC spectra of an uncapped WS$_{2}$ monolayer placed on a $d=240$-nm-thick hBN layer (sample s$_1$) are shown. In panel (b) and (c) the corresponding spectra of an hBN-encapsulated WS$_{2}$ monolayer with a $d=15$-nm-thick (s$_2$) and, respectively, $d=240$-nm-thick (s$_3$) hBN bottom-layer are presented. The PL spectra are excited non-resonantly with laser light of 2.33 eV energy. For all structures, we observe the PL lines of the neutral exciton X and the triplet T$_{\mathrm {T}}$ and singlet T$_{\mathrm {S}}$ trion, as reported in previous publications on WS$_{2}$ monolayers^7,18,24^. Since the exciton energy $E_{\mathrm {X}}$ changes from flake to flake between 2.058 and 2.080 eV, the energy scale of each spectrum is referred to the X energy so that the energy difference $E-E_{\mathrm {X}}$ is shown. The PL spectra in Fig. 1a–c demonstrate that the emission intensity of the X line differs for the samples s$_{1}$, s$_{2}$, and s$_{3}$. It is lowest for the uncapped WS$_{2}$/hBN structure (s$_{1}$) and increases for the hBN-encapsulated W$_{2}$ monolayers gradually with the hBN bottom-layer thickness. This indicates a decreasing electron density in the structures s$_{1}$, s$_{2}$, and s$_{3}$, which in turn is consistent with a decreasing exciton–trion energy splitting in the respective samples.

**Figure 2:**
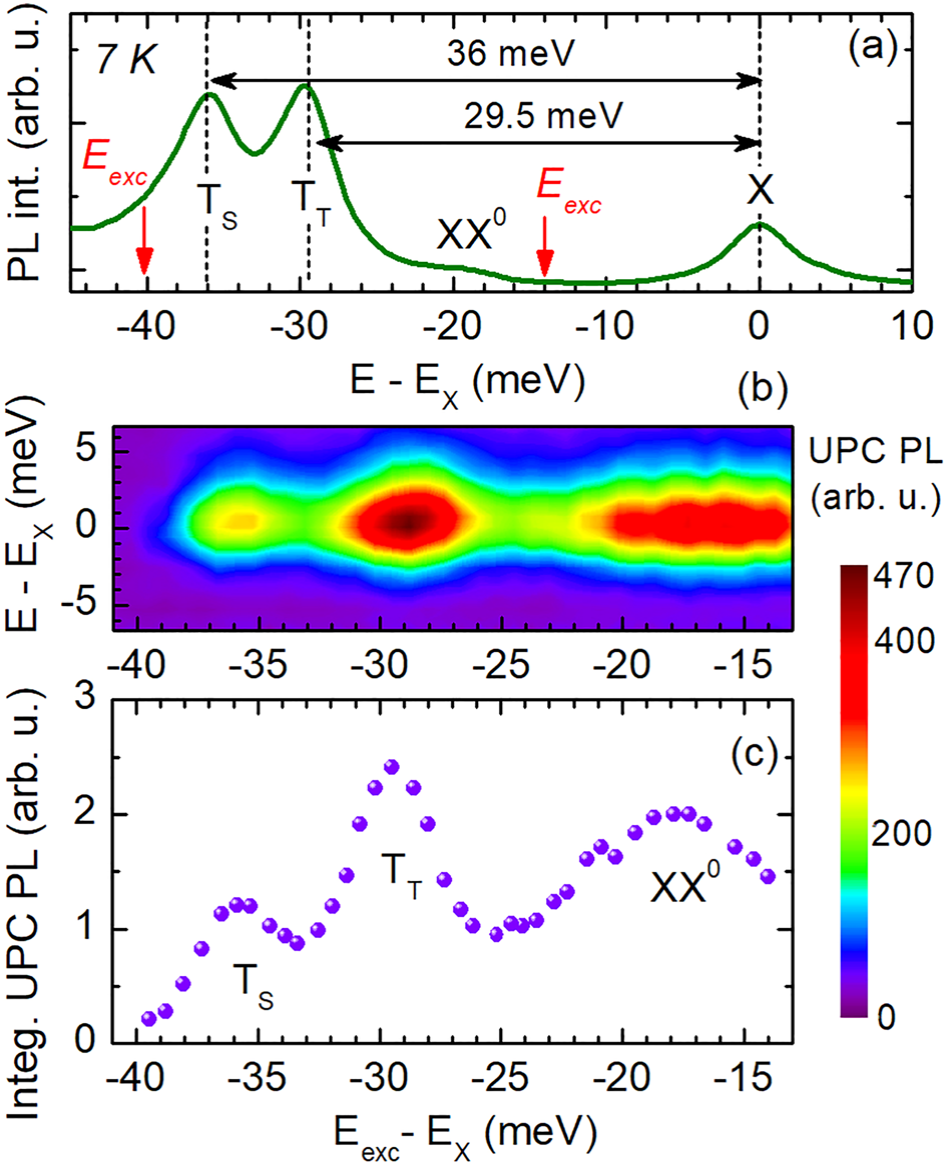
(**a**) PL spectrum of the hBN/WS$_{2}$/hBN heterostructure (s$_3$) at 7 K excited by laser light with 2.33 eV energy. (**b**) Color map of the UPC PLE spectra. (**c**) Integrated UPC PL of the neutral exciton for excitation energies ranging from − 40 to − 14 meV with respect to the X resonance.

As depicted in Fig. 1a, both trions T$_{\mathrm {T}}$ and T$_{\mathrm {S}}$ in the sample s$_{1}$ are red-shifted with respect to X by 35 meV and 41 meV, respectively. In s$_{2}$ and s$_{3}$ these values are reduced relatively by 3 meV and about 5 meV, respectively. The comparison of the exciton–trion peak separation with results recently presented for hBN/WS$_{2}$/hBN heterostructures^24^ allows us to assume that the electron concentration in s$_{1}$ and s$_{2}$ is higher than in s$_{3}$, in which it ranges about (1–1.3) $\times 10^{11}$ cm$^{-2}$ being closely to a charge neutrality. Also, the appearance of the neutral biexciton XX$^{0}$ about 18 meV below X in the PL spectra^25^ attributes the lowest electron density to the sample s$_{3}$. Similar conclusions can be drawn from the complementary RC spectra. The neutral exciton resonance is dominant for all structures; however, as the electron concentration increases, for s$_{2}$ and s$_{3}$, the trion resonances start to emerge in the RC spectra. The significantly higher electron doping in uncapped WS$_{2}$/hBN structures in comparison to hBN-encapsulated (isolated) monolayers may result from laser induced doping effects^7^. Also, defects randomly distributed in the monolayer may act as donors or acceptors leading to a significant *p*- or *n*-doping^26^.

It is worthwhile to emphasize that vdW heterostructures assembled from an hBN-encapsulated WS$_{2}$ monolayer with a relatively low electron doping (close to charge neutrality) allow for observing subtle optical properties of exciton complexes. Hence, they offer the exploration of many-body effects including electron–electron and electron–phonon interactions. Our experiments on exciton upconversion thus focus on sample s$_{3}$.

### Upconverted exciton photoluminescence of hBN/WS$_{2}$/hBN heterostructure

The regular and upconverted PL of the hBN/WS$_2$/hBN vdW heterostructure exhibiting the lowest electron doping (s$_{3}$) recorded at 7 K is demonstrated in Fig. 2. The spectrum shown in Fig. 2a shows the PL lines of different excitonic complexes relative to the X line. The respective energy difference $E-E_{\mathrm {X}}$ is indicated by the *x*-axis.

The UPC PL is excited resonantly by scanning the laser energy from the low-energy flank of the T$_{\mathrm {S}}$ peak to the high-energy flank of the XX$^0$ peak; these limits of the excitation energy $E_{\mathrm {exc}}$ are marked by the red arrows in Fig. 2a,b. The UPC PLE spectra shown in Fig. 2b exhibit the neutral exciton PL line as a function of the excitation energy $E_{\mathrm {exc}}$ detuned from $E_{\mathrm {X}}$. This energy difference is denoted by the UPC energy gain $\Delta E$. The dependence of the integrated exciton UPC PL intensity on the energy gain is depicted in Fig. 2c. It highlights three resonances at the absolute energy gains of 18.0, 29.5, and 36.0 meV. The first resonance matches the spectral position of the biexciton XX$^{0}$, while the other two correspond to the spin-triplet T$_{\mathrm {T}}$ and spin-singlet trion T$_{\mathrm {S}}$, respectively.

**Figure 3:**
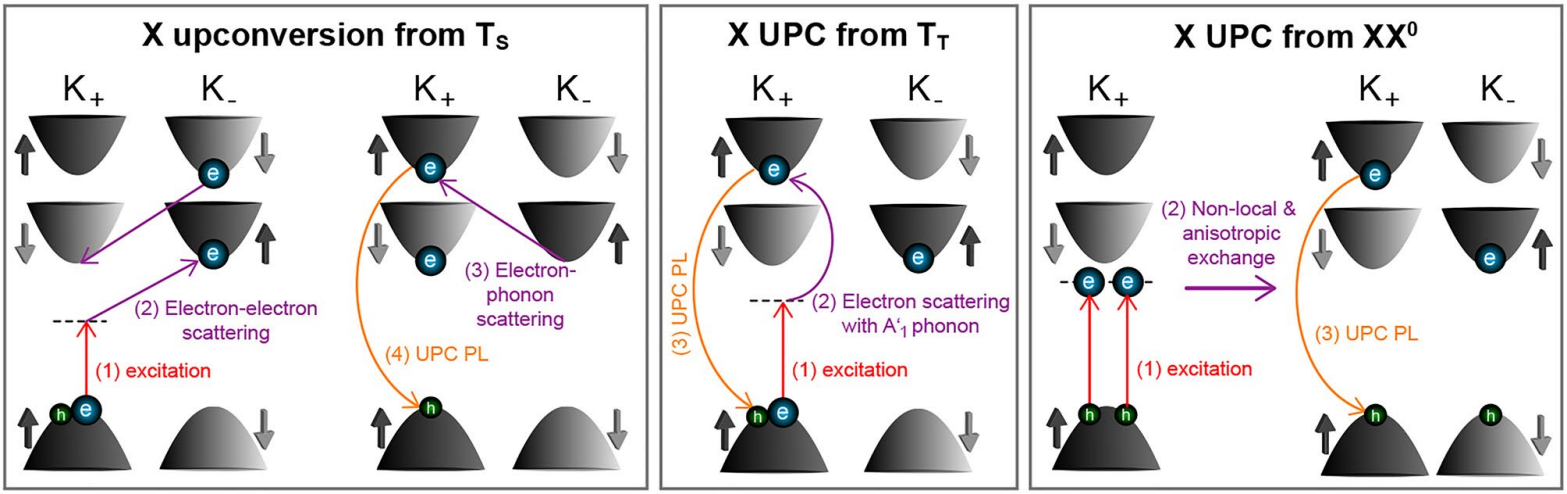
Schematic presentation of the mechanisms of the exciton upconversion involving the spin-singlet and spin-triplet trions as well as the neutral biexciton. The single-particle picture is chosen, where an electron (a hole) is sketched by a blue (green) sphere.

For the exciton emission upconverted from the trion states, we propose the following spin-conserving scattering processes, where we consider a $\sigma ^{+}$ polarized excitation and detection. Initially, for the intravalley singlet trion T$_{\mathrm {S}}$, a spin-down resident electron is at the $K_{-}$ valley. After creating a virtual exciton within the band gap at the $K_{+}$ valley, the spin-up photo-electron is scattered by coupling with the resident electron to the energetically lowest subband at the $K_{-}$ valley, as shown in Fig. 3. The resident electron is in turn scattered to the $K_{+}$ valley under spin conservation. This second stage could also be described in terms of a cooling of the resident electron gas allowing for the electronic scattering. The third stage of the UPC mechanism is established by an electron–phonon coupling shifting the photo-electron to the spin-up subband at the $K_{+}$ valley yielding a carrier configuration similar to that of the intra-valley spin-singlet trion. Finally, the photo-electron recombines with the hole giving rise to $\sigma ^{+}$ polarized bright exciton emission.

The UPC of the exciton emission based on addressing the intervalley triplet trion T$_{\mathrm {T}}$ is likely based on the interaction with a single phonon. The A$'_{1}$ phonon energy fits well with the energy gain of 29.5 meV^11,14,17^. In this resonant phonon scattering process the spin as well as the valley momentum are conserved. At the final stage, one resident electron remains at the spin-up subband of the $K_{-}$ valley, while the spin-up photo-electron as well as the hole at the $K_{+}$ valley recombine resulting in the bright exciton emission.

Interestingly, it has been recently discussed by Ayari et al.^17^ that the contribution of different phonon modes to UPC processes in TMDCs strongly depends on the temperature, dielectric environment and carrier density. The UPC rate of the homopolar mode A$'_{1}$ is independent of the dielectric environment, while for the longitudinal optical phonon E$^{'}$ the strength of the Fröhlich interaction depends on the polarization properties of the encapsulating materials^17,27,28^. The theoretical analysis has also shown^17^ that UPC rates due to E$^{'}$ and A$'_{1}$ phonons become comparable, for a large screening length in hBN-encapsulated W-based monolayers.

Considering the neutral biexciton as real intermediate state for the upconverted X emission, we propose the initial excitation of two virtual excitons at the $K_{+}$ valley; see Fig. 3. Due to a non-local exchange interaction in accordance with Ref. ^29^, the electrons and holes are scattered so that an electron–hole pair is located both at the $K_{+}$ (with spin-up configuration) and $K_{-}$ valley (with spin-down alignment). In addition to that non-local exchange, we consider an anisotropic electron–hole exchange interaction which changes the electron spin while keeping the hole spin unchanged^30^. This anisotropic flip-stop-like exchange interaction is caused by spatially shifted probability densities of the electron and hole at the K$_{-}$ valley. When the centers of their in-plane localization areas do not coincide, the symmetry of this carrier complex is lowered and, in turn, a projection of the angular momentum on the axis defined by the vector linking both centers is not preserved. This symmetry reduction allows for flipping the spin of the electron while leaving that of the hole unchanged. Consequently, a spin-forbidden exciton is given at the $K_{-}$ valley, as illustrated in Fig. 3. The combination of the bright (spin-allowed) and dark (spin-forbidden) excitons residing at the $K_{+}$ and $K_{-}$ valleys, respectively^19,31^, represents the XX$^{0}$. Finally, from this biexcitonic state the electron–hole pair at the $K_{+}$ valley annihilates and causes the experimentally observed enhancement of the exciton emission.

**Figure 4:**
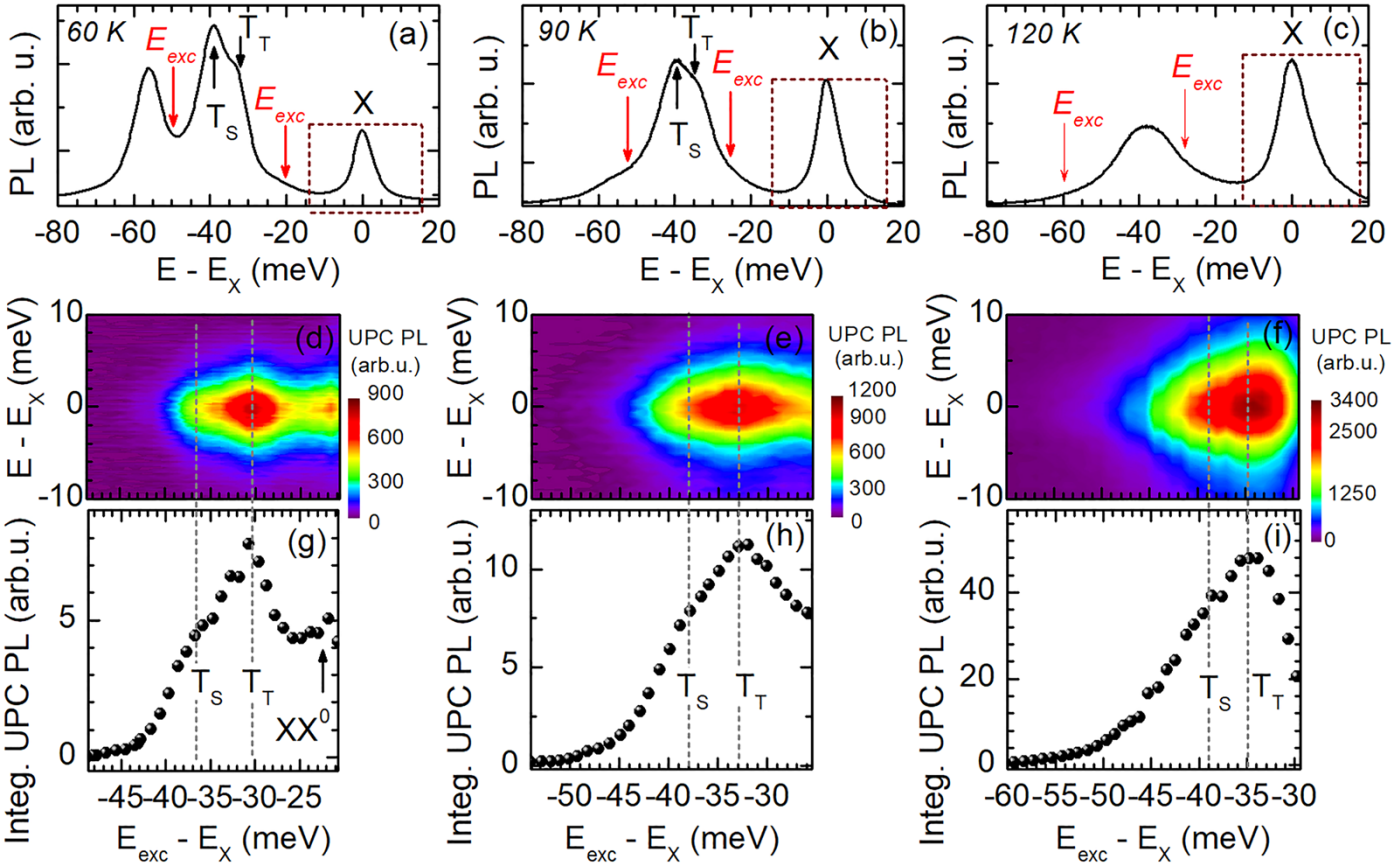
PL spectra of the hBN/WS$_{2}$/hBN monolayer measured at (**a**) 60 K, (**b**) 90 K, and (**c**) 120 K. (**d**–**f**) UPC PL spectra for varying energy gain $\Delta E$ taken at 60 K, 90 K, and 120 K, respectively. (**g**–**i**) Integrated UPC PL of X as function of $\Delta E$ recorded at 60 K, 90 K, and 120 K, respectively.

### Temperature and polarization dependence of the regular and upconverted photoluminescence

**Figure 5:**
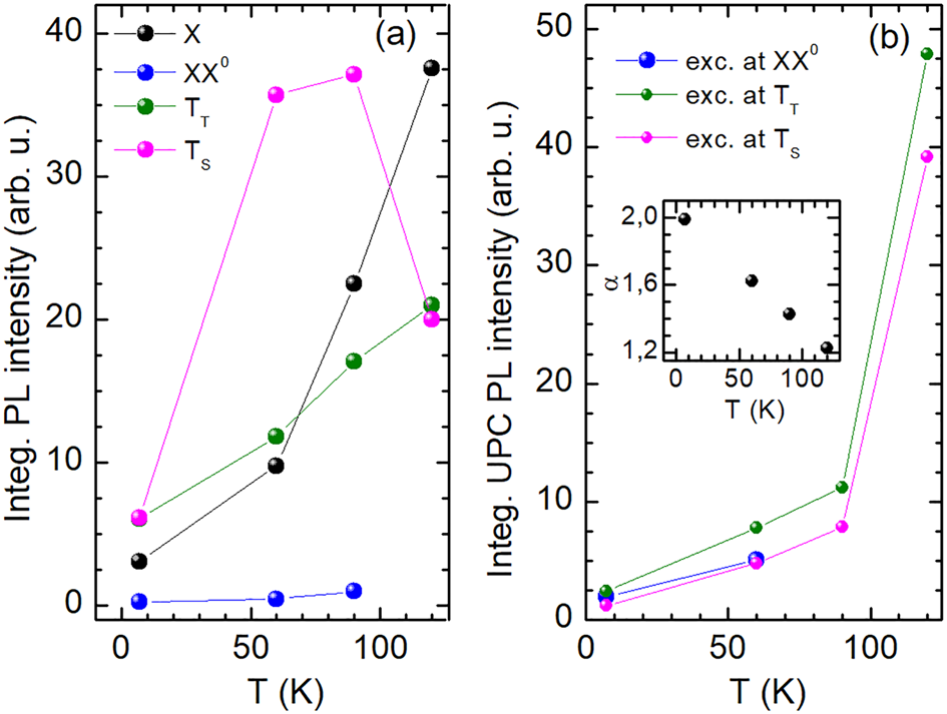
Temperature dependence of (**a**) the integrated PL intensity of the X, XX$^{0}$, T$_{\mathrm {T}}$ and T$_{\mathrm {S}}$ and (**b**) the integrated UPC PL of the X excited at energies of the XX$^{0}$, T$_{\mathrm {T}}$ and T$_{\mathrm {S}}$. The inset of the panel (**b**) shows a temperature evolution of the ratio $\alpha =I^{\mathrm {UPC}}_{\mathrm {T}_{\mathrm {T}}}/I^{\mathrm {UPC}}_{\mathrm {T}_{\mathrm {S}}}$.

The temperature-dependent UPC PLE shall provide insight into an increasing phonon population as well as a thermally enhanced electron scattering. In Fig. 4 the PL spectra, the UPC PLE spectra and the integrated UPC PL of the exciton are shown for the temperatures 60 K, 90 K, and 120 K. For scanning the laser excitation energy between the low-energy flank of the T$_{\mathrm {S}}$ PL line and the high-energy flank of the T$_{\mathrm {T}}$ PL line and detecting the response of the X PL, as marked by the red arrows and dashed rectangle in the panels (a), (b), and (c), we obtain UPC PLE spectra which are presented in the panels (d), (e), and (f). The integrated UPC PL of X recorded at the three different temperatures is depicted in the third row of Fig.  4. The UPC intensity enhancements at the T$_{\mathrm {T}}$ and T$_{\mathrm {S}}$ resonances are distinguishable, independent of the temperatures applied. The UPC resonances are nevertheless thermally broadened which is in agreement with the broadening of the trion PL; see Fig. 4a–c. A thermal broadening is also observed in the integrated UPC PL demonstrated in Fig. 4g–i.

From the fitting of the different exciton PL lines by a combination of Lorentzian curves, we evaluate the integrated intensity of the biexciton and trion PL. Figure 5a summarizes these results showing the temperature dependence of the integrated PL intensity of X, XX$^{0}$, T$_{\mathrm {T}}$, and T$_{\mathrm {S}}$, while in Fig. 5b the integrated PL upconverted from the biexciton and trions to the neutral exciton is demonstrated. As seen in Fig. 5a the integrated X PL intensity (black points) is enhanced with increasing temperature, whereas the integrated XX$^{0}$ emission (blue points) remains nearly constant. The integrated PL intensity of T$_{\mathrm {T}}$ (green points) and T$_{\mathrm {S}}$ (pink points) also increases for rising *T*, whereby the T$_{\mathrm {S}}$ line is dominant up to 90 K. At 120 K the T$_{\mathrm {S}}$ PL weakens and both trion features are broadened. The UPC PL intensities integrated for all initial excitonic states are clearly enhanced with increasing temperature; see Fig. 5b. The most intense upconverted exciton PL $I^{\mathrm {UPC}}_{\mathrm {T}_{\mathrm {T}}}$ is obtained for exciting the triplet trion T$_{\mathrm {T}}$, also in comparison to the upconverted PL $I^{\mathrm {UPC}}_{\mathrm {T}_{\mathrm {S}}}$ for T$_{\mathrm {S}}$ excitation. The latter is presented in the inset of Fig. 5b which includes the temperature evolution of the ratio $\alpha =I^{\mathrm {UPC}}_{\mathrm {T}_{\mathrm {T}}}/I^{\mathrm {UPC}}_{\mathrm {T}_{\mathrm {S}}}$. The ratio slightly decreases with increasing temperature.

The intensities of the regular and upconverted exciton PL in hBN-encapsulated WS$_2$ exhibit a similar temperature-dependent behavior. This is contradictory to the behavior observed in uncapped WS$_{2}$/hBN monolayers^14^, where the UPC PL of the exciton becomes intensified at 120 K and even exceeds the regular PL which practically remains constant with increasing temperature. The thermally induced PL enhancement in the vdW heterostructure studied may be caused by a more efficient non-radiative relaxation of the carriers to the band minima, a thermally induced brightening of dark states and a dissociation of carriers from defects contributing to the excitonic emission. The temperature-dependent growth observed in the exciton UPC efficiency is governed by an increasing phonon population leading to more probable phonon-mediated scattering of the electrons^11,14^. Moreover, at high temperatures the electron–electron scattering within the UPC from the singlet trion is more likely to take place. This thermally more efficient scattering channel explains the enhanced contribution of $I^{\mathrm {UPC}}_{\mathrm {T}_{\mathrm {S}}}$ to $\alpha$ with rising temperature.

**Figure 6:**
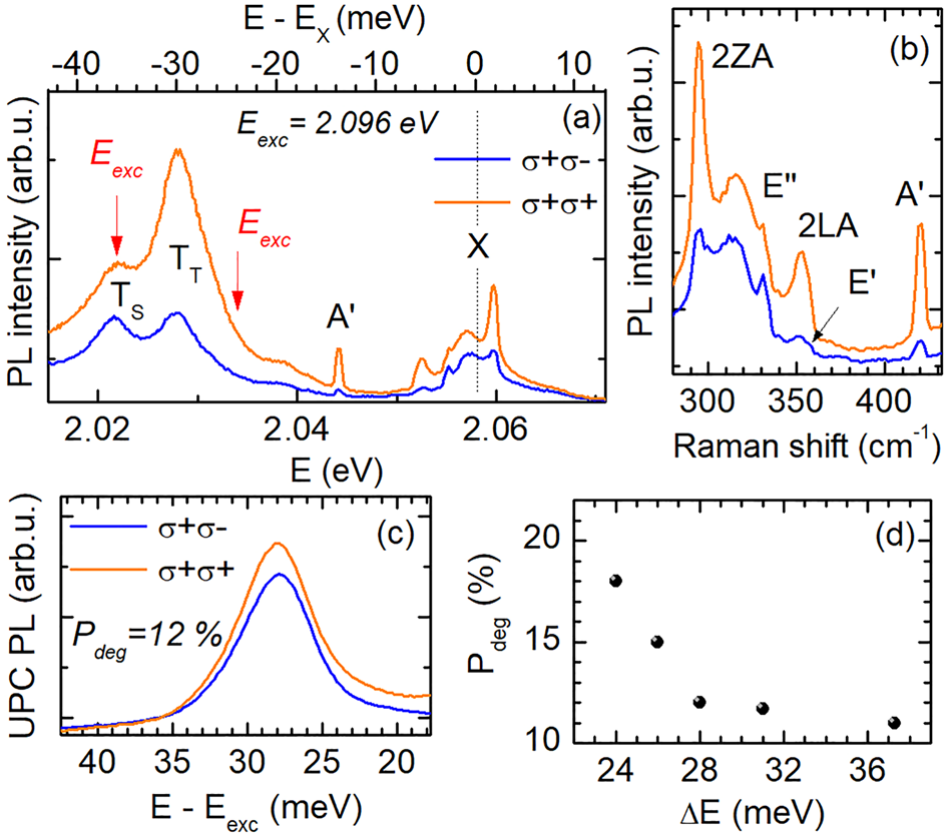
(**a**) Circularly polarized PL spectra for quasi-resonant exciton excitation at 2.096 eV. (**b**) Helicity-resolved Raman scattering spectra excited at 2.096 eV. (**c**) Co- and cross-polarized UPC PL spectra at 7 K, showing an enhancement in the exciton emission at about 28 meV below the X resonance. (**d**) Evolution of $P_{\mathrm {deg}}$ as function of the energy gain $\Delta E$.

Finally, we elucidate the circular polarization properties of the regular and upconverted PL as well as Raman scattering lines performing quasi-resonant and circularly polarized PL and UPC PL experiments. Figure 6a shows PL spectra excited at 2.096 eV and measured at 7 K, for co- and cross-circular polarized configurations. To estimate the circular polarization or, respectively, helicity preservation in the trion PL with respect to the polarization of the exciting laser light, we determine the polarization degree by $P_{\mathrm {deg}} = (I_{\sigma ^{+}\sigma ^{+}} - I_{\sigma ^{+}\sigma ^{-}})/(I_{\sigma ^{+}\sigma ^{+}} + I_{\sigma ^{+}\sigma ^{-}})$, where $\sigma ^{+}\sigma ^{+}$ and $\sigma ^{+}\sigma ^{-}$ indicate the co-circularly and cross-circularly polarized configurations, respectively. The PL of the triplet trion exhibits a polarization degree of almost $59\%$, while, for the T$_{\mathrm {S}}$ line, we obtain $P_{\mathrm {deg}} \approx 22\%$. This is in line with previous results obtained for a WS$_{2}$ monolayer^18,32^. However, it is worth noting that the circular polarization degree of trions varies in different monolayers, since it results from the interplay between different effects, such as scattering processes (radiative and non-radiative types), photo-doping, and free carrier redistribution among the two *K*-valleys^32^. High positive values of the circular polarization degree of the triplet trion are associated with an efficient spin-valley pumping of the resident electrons in a WS$_2$ monolayer using circularly polarized laser light^32^.

In the PL spectra we also distinguish Raman scattering features in the energy range of 2.044–2.062 eV. They are presented in Fig. 6b and are attributed to first- and second-order scattering processes including the A$^{'}$, E$^{'}$, E$^{''}$ modes and 2LA(M) and 2ZA(M) bands, respectively^33^.

Figure 6c depicts circularly polarized UPC PL spectra with a resonance at an energy gain of about 28 meV; the excitation energy was tuned from the T$_{\mathrm {S}}$ peak to the high-energy flank of the T$_{\mathrm {T}}$ peak, as marked by the red arrows in Fig. 6a. The resonance coincides with the spectral position of the intervalley triplet trion T$_{T}$; compare the upper energy scale in Fig. 6a. The exciton PL upconverted from T$_{\mathrm {T}}$ is slightly co-polarized ($P_{\mathrm {deg}} = 12\%$) with the laser light polarization. The high intensities in the co-polarization configuration imply that the UPC is more efficient for resonantly addressing the $K_{+}$ valley. Moreover, the circular polarization degree of the upconverted X emission is decreasing with increasing energy gain from $18\%$ for $\Delta E =24$ meV to $11\%$ for 37 meV, as seen in Fig. 6d. This falling trend, for energetically departing from the trion resonances, corresponds to an increasing probability of scattering processes which change the valley momentum of the carriers. Besides that, $P_{\mathrm {deg}}$ for the UPC PL is slightly lower than values ($21\%$) obtained for regular PL under nearly resonant excitation^34^. This discrepancy is likely caused by a possible scattering of bright excitons into momentum-dark states due to the interaction with *K*-point phonons or electrons. It is also worthwhile to mention that the preservation of the exciton valley polarization, for T$_{\mathrm {T}}$ excitation, is consistent with the polarization properties of the A$'_{1}$ phonon^11^; see also Fig. 6b.

## Discussion

We present temperature-dependent and polarization-resolved PL of the bright neutral exciton upconverted from singlet and triplet negative trions and the neutral biexciton, for a hBN-encapsulated WS$_{2}$ monolayer possessing a relatively low electron doping in the range of (1–1.3) $\times 10^{11}$ cm$^{-2}$. The resonant tuning of the excitation energy through the XX$^{0}$, T$_{\mathrm {T}}$ and T$_{\mathrm {S}}$ states, lying below the neutral exciton, leads to characteristic energy gains of 18.0, 29.5 and 36.0 meV, respectively. These energy gains in the UPC processes are attributed to interactions with resonant optical phonons and, respectively, to a cooling of resident electrons. In the exciton emission upconverted from the trions, spin-conserving electron–phonon and electron–electron scatterings are involved, while the biexciton-mediated UPC is related to a non-local electron–hole exchange followed by an anisotropic electron–hole interaction. The temperature dependence of the upconverted exciton PL intensity demonstrates significant enhancements up to 120 K. They underline both an increasing phonon population giving rise to a high phonon-mediated scattering rate of the electrons as well as a more efficient electron–electron scattering within the UPC involving the singlet trion. Both trions and the neutral biexciton act as real states in the particularly electron- and/or phonon-mediated UPC processes whose circular polarization properties imply that the UPC occurs more efficiently at the $K_{+}$ valley, as the UPC PL has predominantly the same helicity as the exciting laser light. Our results extend the current discussion on exciton–trion and exciton–biexciton couplings in WS$_2$ monolayers as well as the understanding of optical UPC mechanisms in 2D materials.

## Methods

### Bulk WS$_{2}$

The WS$_2$ crystals were grown by the chemical vapor transport (CVT) technique. Prior to the crystal growth, the powdered compounds were prepared from the elements (W: 99.99$\%$, S: 99.999$\%$) by reaction at 1000 $^\circ$C, for 10 days in quartz ampules. The mixture was slowly heated to 1000 $^\circ$C. The chemical transport was achieved with I$_2$ as a transport agent in the amount of about 5 mg/cm$^3$. The growth temperature was changed from 1030 to 980 $^\circ$C with a temperature gradient of 3 $^\circ$C/cm and a growth time of 20 days. The crystals had the shape of thin layered plates with thicknesses and surface areas ranging from 20 to 1000 $\upmu$m and 20 to 100 mm$^2$, respectively.

### Monolayer WS$_2$

**Figure 7:**
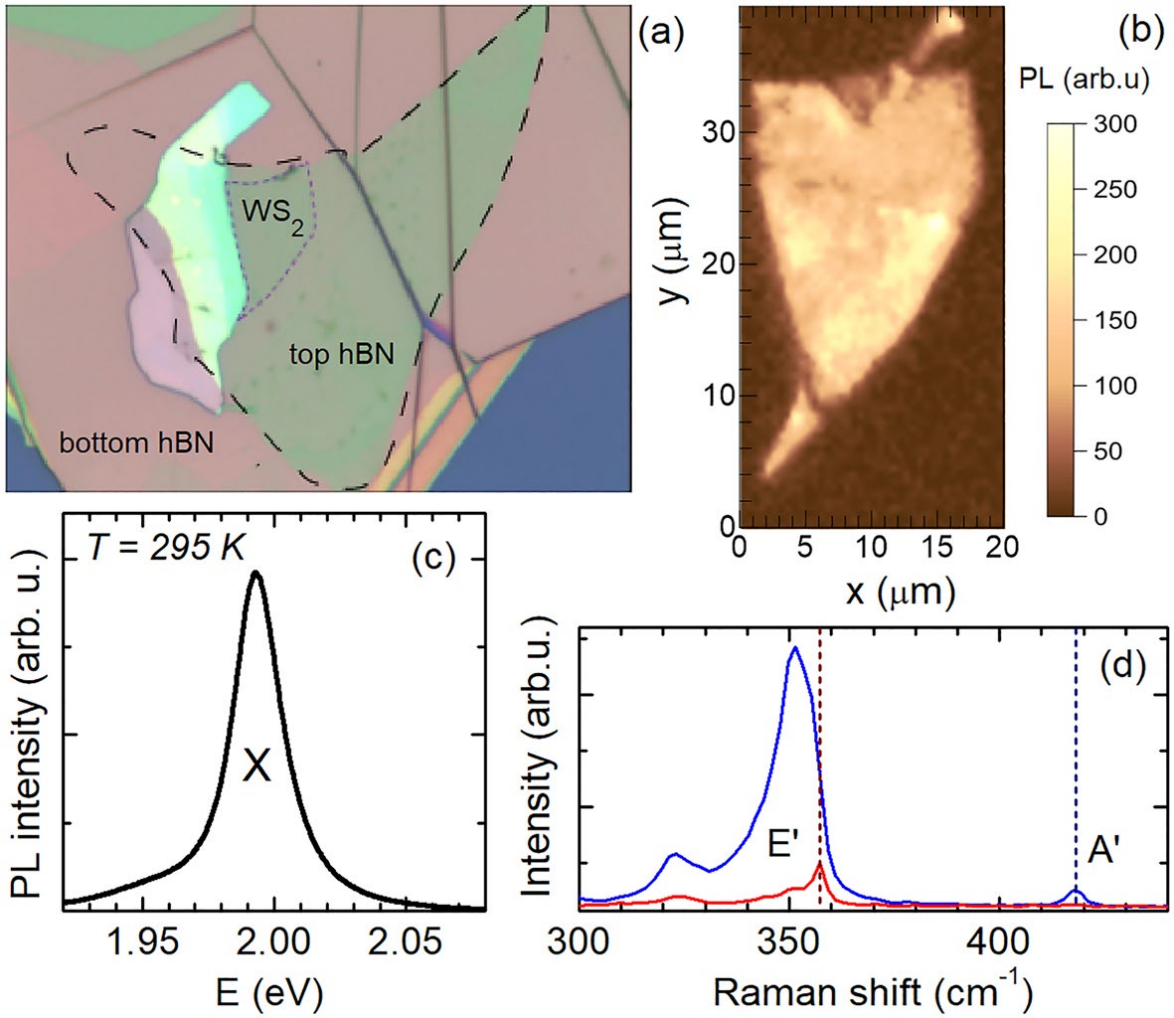
(**a**) Optical microscope contrast image of the hBN/WS$_2$/hBN/SiO$_2$/Si heterostructure. (**b**) PL intensity map across the WS$_2$ monolayer studied. (**c**) Exemplary PL spectrum showing the emission of the neutral exciton, $E_{\mathrm {exc}}= 2.33$ eV. (**d**) Room-temperature Raman scattering spectra including the $E^{'}$ and $A^{'}$ optical phonons.

We prepared hBN-encapsulated WS$_2$ and uncapped WS$_2$/hBN heterostructures using high-purity hexagonal boron nitride (hBN) and a target Si substrate with 300-nm-thin SiO$_2$. The WS$_2$ monolayers were mechanically exfoliated from the aforedescribed bulk crystals. For this purpose, the deterministic all-dry stamping method was applied^35^. The WS$_2$ monolayer and the hBN crystals were first exfoliated on a flexible polydimethylsiloxane gel-film stamp which was rigidly attached to a glass slide. The thicknesses of the hBN flakes were $\sim$ 15 nm and $\sim$ 240 nm, for the bottom layer, and up to $\sim$ 5 nm, for the top layer. During the transfer process the substrate and the stamp were inspected using an optical microscope equipped with an *xyz* stage. The application of a long-working distance objective enables us to locate and deterministically transfer selected flakes to the substrate by carefully bringing the stamp in contact with the substrate. After each transfer step, the samples were heated to $\sim$ 180 $^\circ$C in air for 20 minutes. After adding the last layer to the stacked samples, an annealing at $\sim$ 200 $^\circ$C was performed in air for 2 h. Figure 7a presents an optical microscope contrast image of the hBN/WS$_2$/hBN/SiO$_2$/Si heterostructure with the thickness of the hBN bottom-layer $d=240$ nm. The monolayer character of the WS$_2$ flake was confirmed by PL and Raman scattering experiments performed at room temperature under ambient conditions. Figure 7b shows a PL intensity map of the WS$_2$ monolayer recorded at the neutral exciton energy of 1.995 eV. A typical PL spectrum including the exciton line is demonstrated in Fig. 7c. Linearly polarized Raman scattering spectra depicted in Fig. 7d clearly identify the $E^{'}$ and $A^{'}$ optical phonons located at 357.5 cm$^{-1}$ (44 meV) and 418 cm$^{-1}$ (52 meV), respectively.

### Experimental setup

The samples were mounted on the cold finger of a vibration-isolated closed-circle cryostat which allowed for temperature variations between 7 and 300 K. The PL was excited by the second harmonic (532 nm, 2.33 eV) of a continuous-wave single-mode Nd:YAG laser. The UPC PLE was excited by a continuous-wave dye laser equipped with DCM whose emission was tuned in the range from 602 (2.06 eV) to 650 (1.91 eV). The laser beam was focused on the sample under normal incidence using a long-working distance microscope objective with 50$\times$ magnification. The diameter of the excitation spot was about 1 $\upmu$m. The emission was collected by the same microscope objective, was dispersed by a monochromator with 0.5 m focal length and 600 lines/mm grating and was detected by a Peltier-cooled charged couple device Si-camera. The Raman spectra were measured in the backscattering geometry using either 1800 or 600 lines/mm gratings. The impact of elastically scattered light on the spectra was reduced by a set of spectrally appropriate edge filters.

## Data Availability

The data sets used and/or analyzed during the current study are available from the corresponding author on reasonable request.
